# The comparison data forest: A new comparison data approach to determine the number of factors in exploratory factor analysis

**DOI:** 10.3758/s13428-023-02122-4

**Published:** 2023-06-15

**Authors:** David Goretzko, John Ruscio

**Affiliations:** 1 LMU Munich, Department of Psychology, Munich, Germany; 2 Utrecht University, Department of Methodology and Statistics, Padualaan 14, 3584 Utrecht, CH The Netherlands; 3 The College of New Jersey, Ewing, USA

**Keywords:** Exploratory factor analysis, Comparison data, Factor retention, Number of factors, Factor forest, Machine learning

## Abstract

Developing psychological assessment instruments often involves exploratory factor analyses, during which one must determine the number of factors to retain. Several factor-retention criteria have emerged that can infer this number from empirical data. Most recently, simulation-based procedures like the comparison data approach have shown the most accurate estimation of dimensionality. The factor forest, an approach combining extensive data simulation and machine learning modeling, showed even higher accuracy across various common data conditions. Because this approach is very computationally costly, we combine the factor forest and the comparison data approach to present the comparison data forest. In an evaluation study, we compared this new method with the common comparison data approach and identified optimal parameter settings for both methods given various data conditions. The new comparison data forest approach achieved slightly higher overall accuracy, though there were some important differences under certain data conditions. The CD approach tended to underfactor and the CDF tended to overfactor, and their results were also complementary in that for the 81.7% of instances when they identified the same number of factors, these results were correct 96.6% of the time.

## Introduction

Psychological research relies heavily on latent variables that are unobservable and therefore measured using manifest indicators. Exploratory factor analysis (EFA) models the links between one or more latent variables, or factors, and a set of manifest indicators. Hence, it is frequently used in the development of psychological scales and assessment tools. When performing an EFA, several methodological decisions have to be made by the researcher (see Fabrigar, Wegener, MacCallum, & Strahan, 1999; Goretzko, Pham, & Bühner, 2019), and determining the number of factors seems to be the most difficult (Henson & Roberts, 2006; Zwick & Velicer, 1986). That said, retaining the correct number of factors should be of utmost importance, especially when an EFA is used to construct or validate an instrument used for psychological assessment. In cases where too few factors are extracted, theoretically interesting subscales of psychological constructs could be missed, while overfactoring (i.e., extracting too many factors) may lead to blurred concepts with an artificially increased number of subfacets. A clear conceptualization of the respective latent variables and an accurate factor retention are essential prerequisites for trustworthy psychological assessment and valid measurement instruments for use in clinical settings. Thus, researchers who develop these instruments have to be very careful when determining the number of factors to retain in EFA.

Since EFA is often used in contexts in which theoretical guidance about dimensionality is not available, the number of factors has to be estimated from the data. Traditional approaches like the Kaiser–Guttman rule (Kaiser, 1960) – also known as the eigenvalue-greater-one rule – or the scree test (Cattell, 1966) are heuristic rules based on the empirical eigenvalue distribution. Parallel analysis (Horn, 1965), as well as more modern approaches like comparison data (CD, Ruscio & Roche, 2012), make use of the larger computational resources now available to simulate reference eigenvalues based on characteristics of the empirical data. Another new and promising method, the empirical Kaiser criterion (EKC, Braeken & Van Assen, 2017) also compares the empirical eigenvalues with reference values that are a function of the variables-to-sample-size ratio and a correction term for the variance that previous factors have already accounted for.

Focusing on the eigenvalue distribution to determine the number of factors makes sense because eigenvalues of a correlation matrix are directly linked to the explained variance of a component in principal component analysis (PCA). However, as Braeken and Van Assen (2017) point out, sampling error deteriorates the informational value of the empirical eigenvalue distribution. Hence, Goretzko and Bühner (2020) developed a new factor retention criterion that is based on an extensive simulation step and a subsequent step where a machine learning model is trained to predict the number of factors based on the empirical eigenvalues and additional data characteristics. Their evaluation study showed that this new approach, called a factor forest, is superior to common retention criteria reaching almost perfect accuracy over a broad range of conditions. However, because the creation of a factor forest is computationally costly, pre-trained machine learning models would have to be provided for practitioners (Goretzko & Bühner, 2022). Accordingly, if the empirical data do not meet the distributional assumptions of the training data used to generate these pre-trained models, their applicability might be impaired and a new model would have to be trained using newly simulated training data. The complete process would be very time-consuming.

To circumvent this issue, we combine the factor forest with the CD approach that is able to adapt to the empirical data and does not require strong distributional assumptions. In an extensive simulation study, we compare this new combined approach, called a comparison data forest (CDF), with the traditional CD approach. We derive recommendations for the hyperparameter settings of both factor retention criteria as well as suggestions for when to use which approach.

### Comparison data – Using reference eigenvalues to determine the number of factors

Ruscio and Roche (2012) introduced the CD approach as a way to improve what was then widely considered the best available method for determining the number of factors to retain in EFA, namely parallel analysis. When using parallel analysis, one generates a large number of random data sets with the same number of cases and variables as the empirical data, but in which the data are normally distributed, uncorrelated variables. One estimates the number of factors to retain as the number of eigenvalues for the empirical data that exceed the mean eigenvalues for the parallel analysis of all samples of random data. The parallel analysis approach works comparably well because it takes into account sampling error (Turner, 1998). Simulation studies (e.g., Auerswald & Moshagen, 2019; Zwick & Velicer, 1986) usually show that parallel analysis is among the most accurate factor retention criteria.

The CD approach also generates a large number of data sets in order to obtain reference eigenvalues, but it differs from parallel analysis in three ways. First, whereas the random data in parallel analysis are normally distributed^1^, the CD approach reproduces each empirical indicator’s distribution by using bootstrap methods (Efron & Tibshirani, 1993). Second, whereas the random data in parallel analysis are uncorrelated, the CD approach reproduces the indicator correlation matrix. Third, whereas parallel analysis provides one set of reference eigenvalues, the CD approach provides multiple sets of reference eigenvalues by incrementing the number of factors used to reproduce the indicator correlation matrix. The first population of comparison data is generated using one factor, many random samples are drawn from this population, and a set of reference eigenvalues is obtained in order to calculate their fit to those of the empirical data. Next, a new population of comparison data is generated using two factors, random samples are drawn, reference eigenvalues are obtained, and fit is calculated. Goodness of fit is described by the eigenvalues’ root mean squared residuals (RMSR) where the residuals are defined as the difference between an eigenvalue and its corresponding reference eigenvalue (i.e., the eigenvalue of a comparison data set). This iterative process of increasing the number of factors used to reproduce the indicator correlation matrix continues until fit fails to improve significantly (i.e., until the RMSR values of the k-factor solution are not deemed significantly higher on average than those of the (k+1)-factor solution). For example, if using three factors fails to improve fit relative to what was observed using two factors, this suggests that one should retain only two factors. In their evaluation study, Ruscio and Roche (2012) found that the CD approach outperformed parallel analysis, as well as the many other techniques (Kaiser–Guttman rule, optimal coordinates, the minimum average partial test, AIC, BIC, and sequential $\chi ^2$ tests) they tested, across a wide range of challenging data conditions.

### The factor forest – Using machine learning models to determine the number of factors

Goretzko and Bühner (2020) proposed a new approach to factor retention that makes use of the predictive power of machine learning algorithms. The first step of their approach is to simulate numerous data sets with a known factorial structure that cover all important data conditions of the application context^2^. Data characteristics (the predictor variables which are called features in the context of machine learning applications) that are relevant in the factor retention process are then extracted for each simulated data set (inter alia eigenvalues and matrix norms of the correlation matrix, the sample size, and the number of manifest variables). As described above, the eigenvalues of the empirical correlation matrix are directly related to the variance explained by the respective component in PCA (equivalently, eigenvalues of a reduced correlation matrix with communalities in the diagonal are indicative of the variance explained by the respective factor in an EFA model). Braeken and Van Assen (2017) also consider the size of previous eigenvalues and the sample size when calculating the reference eigenvalues for their criterion, which is why the latter and features that describe the explained variance are also included in the feature set. Furthermore, since EFA can simply be described as a decomposition of the manifest correlation matrix, other features that describe the “size” and composition of the correlation matrix are calculated. One example is the Gini coefficient (Gini, 1921) which is usually used to quantify inequalities in distributions. In this context, it can be used to assess the inequality of all bivariate manifest correlations, since if unidimensionality holds, all correlations should be similar and the more latent factors, the more clusters can be found in the correlation matrix. All features are described more thoroughly in the original article by Goretzko and Bühner (2020).

These features and the known number of factors are then stored as the columns in one combined training data set, which contains one row for each data set simulated in the first step (i.e., each simulated data set is one observation in the training data). A machine learning model is trained^3^ to predict the number of factors using the extracted features as independent variables. Training a machine learning model means that the multidimensional link between the features (data characteristics) and the number of underlying factors is learned based on the fully labeled training data. In other words, the relationship between data characteristics that can be observed for every empirical data set and the number of underlying factors is statistically modeled and reflected by the complex model structure of the trained model. To validate this model, it must be successfully evaluated using a new test sample of simulated data sets. Once validated, it can be used to determine (i.e., to predict) the number of factors on new samples of empirical data.

### The comparison data forest – A combined approach

In this paper, we want to evaluate whether the machine learning modeling approach can be used within the CD framework. We call the new, combined approach the *comparison data forest* (CDF) as it is a combination of CD and the factor forest, or rather a similar random forest implementation (see the pseudo-code below). For CDF, populations with a known factorial structure are simulated as described for the original CD approach using the *GenData* function provided by Ruscio and Roche (2012). *GenData* is an iterative algorithm that aims at finding a k-factor solution that best reproduces the empirical correlation matrix assuming normally distributed latent factors and an unrotated solution (i.e., orthogonal factors and potentially cross-loadings), but taking into account skewed item distributions contrary to parallel analysis. Specifically, populations ranging from 1 to $k_{max}$ factors are simulated and then $N_{rep}$ samples (each with the same sample size as the empirical data set) are drawn from each population. Several features (e.g., eigenvalues and matrix norms)^4^ are calculated for these $N_{rep} \times k_{max}$ comparison data sets and used to train a machine learning model. We used the feature set suggested by Goretzko and Bühner (2020) and chose a random forest, as implemented in the *ranger* package (Wright & Ziegler, 2017), as the machine learning modeling method due to its predictive power and relatively low computational costs. To further reduce the computational costs of this approach, we relied on the well-established default settings of the random forest in the *ranger* package (Wright & Ziegler, 2017) – namely, setting the number of trees to 500 and using the (rounded down) square root of the number of features for each split ($m_{try} = \lfloor \sqrt{p} \rfloor$) as suggested by Breiman (1999). Subsequently, the trained model can be used to predict the number of factors to retain for an empirical data set using the same features as independent variables (i.e., the same features have to be calculated for the empirical data set).
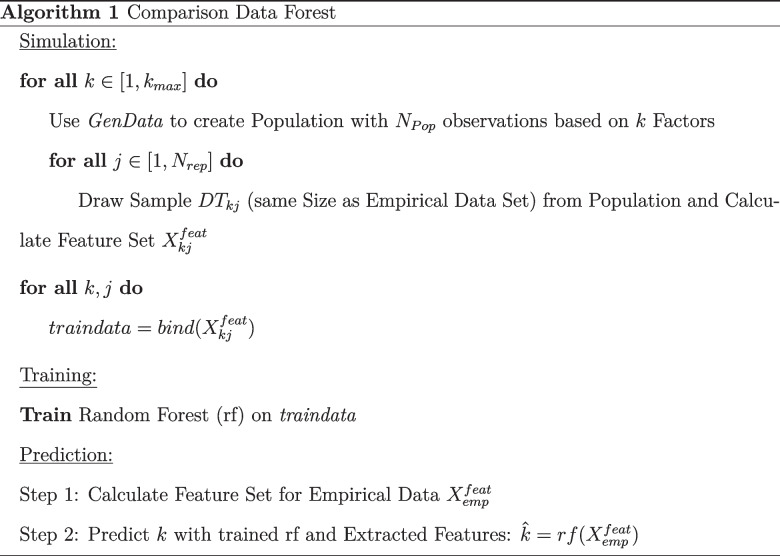


### Hyperparameters of the comparison data approaches

Both CD and CDF have hyperparameters that influence how each method performs and how computationally costly the respective approach is. When using the initial CD approach, researchers have to set three parameters – the significance level for the internal Mann–Whitney *U* test ($\alpha$), the size of the population $N_{population}$ that is simulated for each factor solution and the number of comparison data sets that are drawn from each of these populations $N_{rep}$. While the impact of $\alpha$ appears to be quite clear – higher values increase the statistical power of the test, but also increase the probability for a Type I error, therefore yielding a stronger tendency of overfactoring (and vice versa for smaller values) – both the population size $N_{population}$ and $N_{rep}$ have a less clear influence on CD. In both cases (CD and CDF), higher values seem beneficial as they promise less randomness in the comparison data sets (i.e., a reduced sampling error), but there is obviously a limit in usefulness when increasing $N_{rep}$ and $N_{population}$ (especially, since increasing these numbers will increase computational costs as well). When choosing $N_{population}$, the sample size of the empirical data set has to be considered as well, since all comparison data sets that are drawn from the simulated populations have the same size as the empirical data set. Accordingly, samples that are much larger than those considered in this paper may call for greater population sizes $N_{population}$. Since machine learning models “learn” patterns from large numbers of examples, the number of comparison data sets $N_{rep}$ that yields a good performance might actually be higher for CDF than for CD. While $\alpha$ is no longer a hyperparameter for CDF, the new approach has additional hyperparameters that determine how the internal machine learning model (i.e., the random forest in this case) actually looks like. For a random forest, the number of decision trees, the tree depth or the proportion of variables considered for each split are the most important hyperparameters. Contrary to other machine learning algorithms, random forests perform comparably well with default hyperparameters (e.g., Probst, Wright, & Boulesteix, 2019) and are therefore considered a good “off-the-shelf” option that does not require extensive tuning (e.g., Sterner, Goretzko, & Pargent, 2021). In our implementation, we set the number of trees to 500 and the number of variables considered for each split to the (rounded down) square root of the number of features (Breiman, 2001).

### Aim of the study

In this paper, we want to find optimal default hyperparameters for both comparison data approaches (CD and CDF) and evaluate their performance under various data conditions. Hence, the aim of this study is to establish a new variant of comparison data-based factor retention and to compare it to the initial CD method. For sake of clarity and to save computational costs, we do not expand the simulation study to the previously described factor forest approach^5^.

## Methods

To evaluate under which conditions CDF is a useful new approach to determine the number of factors in EFA and to find appropriate default values for $N_{rep}$ and $N_{population}$, we simulated multivariate normal data using the *mvtnorm* package (Genz et al., 2018) for various data conditions. We varied the true number of factors $k \in \{1,3,5\}$, the sample size $N \in \{250,500,1000\}$, the number of variables per factor $vpf \in \{4,7\}$, the inter-factor correlation $\rho \in \{0,0.2,0.5\}$, as well as the loading magnitudes of primary and cross-loadings according to the simulation settings of Goretzko and Bühner (2020). Standardized primary loadings were sampled from three categories (small: [0.35, 0.5], medium: [0.5, 0.65] and large [0.65, 0.8]) and cross-loadings were sampled/selected from three categories (zero cross-loadings, small: [0, 0.1], and medium: [0.1, 0.2]). In total, we evaluated 372^6^ data conditions with 500 replications each.

**Table 1:** Overall accuracy of CD with different parameter settings

$\alpha$	$N_{rep} = 250$	$N_{rep} = 500$	$N_{rep} = 1000$	$N_{rep} = 2000$
.05	0.843	0.848	0.850	0.849
.10	0.836	0.842	0.844	0.844
.20	0.818	0.826	0.832	0.833
.30	0.797	0.813	0.819	0.822

*Note.* $\alpha$ is significance level used in the internal Mann–Whitney *U* test in the CD approach, while $N_{rep}$ denotes the number of replications or comparison data sets

We analyzed the simulated data with common CD (using the R code provided by Ruscio & Roche, 2012) varying the parameters $\alpha \in \{0.05, 0.1, 0.2, 0.3\}$ and $N_{rep} \in \{250, 500, 1000, 2000\}$ and the new CDF approach with five different parameter settings for $N_{rep}$ and two values for $N_{population}$ ($n_{rep} \in \{100,1000,2000,4000,5000\}$ and $N_{population} \in \{10000,25000\}$). To assess the performance of CD and CDF in comparison to non-simulation-based factor retention criteria, we also calculated the EKC for each simulated data set.

### Data analysis

We used R (Version 4.2.2; R Core Team, 2020) and the R-packages *batchtools* (Bischl, Lang, Mersmann, Rahnenführer, & Weihs, 2015; Version 0.9.15; Lang, Bischl, & Surmann, 2017), *dae* (Version 3.2.13; Brien, 2020), *data.table* (Version 1.14.6; Dowle & Srinivasan, 2019), *ggplot2* (Version 3.4.0; Wickham, 2016), *mvtnorm* (Version 1.1.3; Genz & Bretz, 2009), *papaja* (Version 0.1.1; Aust & Barth, 2020), and *tinylabels* (Version 0.2.3; Barth, 2022) for the data simulation and all our analyses as well as writing the manuscript. For each data condition and each parameter setting of both methods, we calculated the accuracy of the factor retention process (i.e., the relative frequency of the correctly determined number of factors) as well as bias (i.e., the relative frequency and magnitude of over- or underfactoring). The code for our simulation study as well as the implementation of the comparison data forest can be found in the electronic supplementary material.

## Results

### Comparison data

Table 1 shows the overall accuracy of CD for all combinations of evaluated parameter values. Averaged over all conditions, CD attained the highest overall accuracy with a comparably low significance level $\alpha$ and a large value of comparison data sets $N_{rep}$. However, differences were very small, especially with $\alpha \le .10$. Interestingly, the lower $\alpha$ was, the more biased the estimation (average bias for $\alpha =.05$: $-$0.26 and for $\alpha =.30$: $-$0.17). Put differently, CD tended to underfactor with all parameter settings, and this tendency that was stronger with low $\alpha$-levels (on average CD suggested too few factors in 13.10% of the cases when $\alpha =.05$ compared to 11.90% when $\alpha =.30$). Given that the criterion for advancing to a larger number of factors is significant improvement in fit, and that using a lower $\alpha$-level makes it more difficult to attain statistical significance, it is not surprising that underfactoring was more common with lower $\alpha$-levels.

**Fig. 1:**
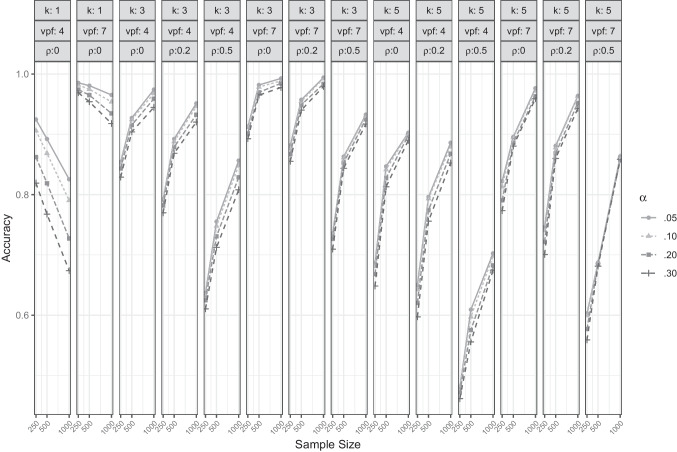
Accuracy of CD for different sample sizes, $\alpha$-levels, numbers of factors (k), variables per factor (vpf) and between-factor correlations ($\rho$)

**Table 2:** Overall accuracy of CDF with different parameter settings

$N_{population}$	$N_{rep} = 100$	$N_{rep} = 1000$	$N_{rep} = 2000$	$N_{rep} = 4000$	$N_{rep} = 5000$
10000	0.821	0.858	0.860	0.863	0.863
25000	0.818	0.856	0.860	0.862	0.862

*Note.* $N_{population}$ is the population size within the CDF approach, while $N_{rep}$ denotes the number of replications or comparison data sets that are drawn per factor solution

Figure 1 presents the accuracy of CD in greater detail for different sample sizes, numbers of factors, variables per factor, and between-factor correlations. The differences between the four $\alpha$-level settings were rather small, though in single-factor conditions a smaller $\alpha$ seems to be clearly favorable. Oddly, when $k=1$, CD showed a higher accuracy with smaller sample sizes; for $k \ge 3$ larger samples yielded higher accuracies. Higher amounts of overdetermination (more variables per factor), as well as smaller between-factor correlations, fostered more accurate factor retention.

**Fig. 2:**
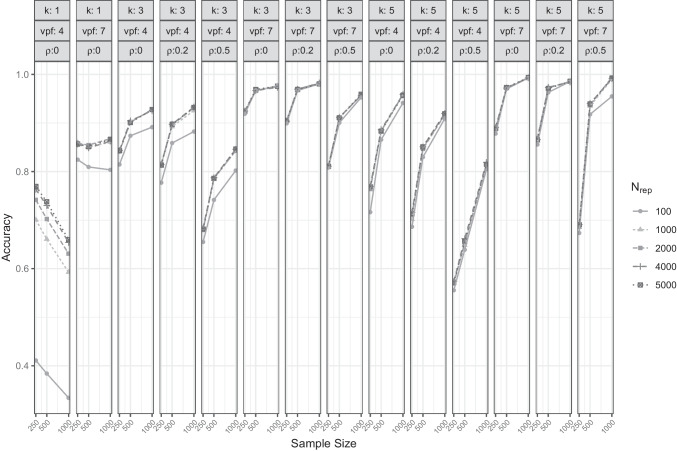
Accuracy of CDF for different sample sizes, $N_{rep}$ values, number of factors (k), variables per factor (vpf) and between-factor correlations ($\rho$)

**Table 3:** Bias of CDF with different parameter settings

$N_{population}$	$N_{rep} = 100$	$N_{rep} = 1000$	$N_{rep} = 2000$	$N_{rep} = 4000$	$N_{rep} = 5000$
10000	0.069	0.018	0.013	0.011	0.010
25000	0.090	0.035	0.028	0.025	0.025

*Note.* $N_{population}$ is the population size within the CDF approach, while $N_{rep}$ denotes the number of replications or comparison data sets that are drawn per factor solution

**Table 4:** Accuracy and Bias of CD and CDF with selected parameter settings (EKC as baseline)

Method	$Acc_{1}$	$Bias_{1}$	$Acc_{3}$	$Bias_{3}$	$Acc_{5}$	$Bias_{5}$
$CD_{.30/2000}$	0.852	0.177	0.871	$-$0.104	0.761	$-$0.354
$CD_{.05/1000}$	0.924	0.079	0.886	$-$0.144	0.787	$-$0.466
$CDF_{5000/10000}$	0.783	0.428	0.895	0.004	0.857	$-$0.132
EKC	0.998	0.002	0.852	$-$0.216	0.750	$-$0.635

*Note.* $CD_{.30/2000}$ stands for the classical comparison data approach with $\alpha =.30$ and 2000 comparison data sets per factor solution. $CDF_{5000/10000}$ stands for the comparison data forest with 5000 comparison data sets per factor solution and a population size of 10000. EKC is the empirical Kaiser criterion. $Acc_1$ is the accuracy in conditions with one true factor, $Acc_3$ and $Acc_5$ the accuracy in conditions with three and five factors respectively. $Bias_1$ shows the bias of each method in conditions with one underlying factor, $Bias_3$ and $Bias_5$ the bias in conditions with three and five underlying factors accordingly

### Comparison data forest

In Table 2, the overall accuracy of the new CDF approach is displayed for the parameter values of $N_{population}$ (number of observations in each simulated population) and $N_{rep}$ (number of comparison data sets simulated for each factor solution). As expected, the number of comparison data sets drawn from each simulated population ($N_{rep}$) was positively associated with the overall accuracy of the approach. Increasing this number from 100 to 1000 boosted the performance by 3.7 percentage points, but increasing it further led to much smaller improvement (i.e., going all the way to $N_{rep} = 5000$ only yielded an additional 0.5 percentage point improvement). Increasing the size of the simulated populations from 10, 000 to 25, 000 did not improve accuracy; in fact, accuracy was lower by up to 0.33 percentage points. Contrary to the classical CD approach, the CDF showed a rather small but positive bias, which means a small tendency for overfactoring (i.e., extracting too many factors). Table 3 displays the average bias of the CDF approach given different parameter settings. CDF with $N_{rep} = 100$ showed a substantial tendency to overfactor (it suggested too many factors in 10.81% of the cases), with an overall bias of 0.07 when $N_{population} = 10,000$ and 0.09 when $N_{population} = 25,000$. In comparison to CD, this average deviation from the true number of factors was rather small, though (bias between $-$0.292 and $-$0.168).

Figure 2 presents the accuracy of CDF in greater detail for varying $N_{rep}$-settings against the sample sizes given the different numbers of factors, variables per factor, and between-factor correlations. For most conditions, there was little to no difference in performance with regard to different values of $N_{rep}$, though $N_{rep} = 100$ was an exception in that accuracy was notably lower in conditions with $k=3$ and little overdetermination (variables per factor $vpf = 4$) as well as conditions with $k =5$, high between-factor correlations ($\rho = 0.5$) and greater sample sizes. For $k \ge 3$, the impact of $N_{rep}$ was negligible (if one excludes $N_{rep} = 100$) – only a minor tendency that smaller values (i.e., $N_{rep} = 1000$) could be preferable in cases with $k = 3$ could be identified.

**Fig. 3:**
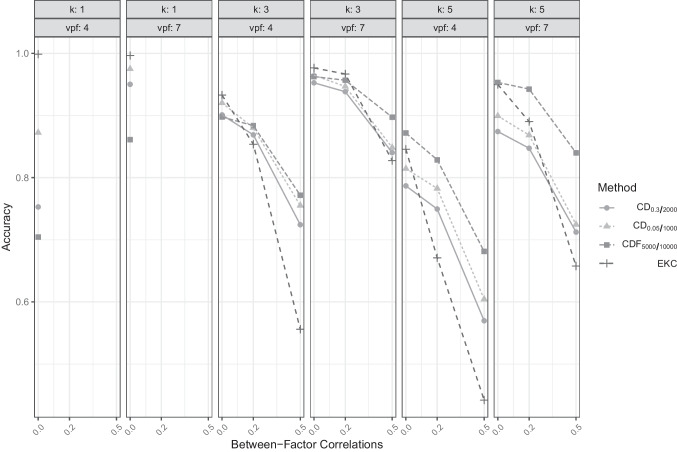
Comparison of CD and CDF with selected hyperparameters (and EKC as a baseline) across conditions with different between-factor correlations ($\rho$), variables per factor (*vpf*) and numbers of factors (*k*)

However, in single-factor conditions ($k = 1$), immense differences in accuracy were present. In conditions with very few manifest variables, CDF struggled to correctly identify unidimensionality. The tendency to overfactor was more severe the smaller $N_{rep}$ and the greater the sample size (bias of CDF with $N_{rep} = 100$ and a sample size of 1000 was 1.32; bias with $N_{rep} = 5000$ and a sample size of 250 was 0.46). Even though larger sample sizes were associated with lower accuracy in these special conditions, more generally an increase in the number of observations was related to a more accurate factor retention (comparable to the results of CD). As expected, a higher rate of overdetermination (i.e., a higher variables-per-factor ratio) as well as smaller between-factor correlations were beneficial for the factor retention process.

### Comparison of CD and CDF

For a detailed comparison of the general approaches, we focus on the hyperparameter settings that yielded the highest overall accuracy (CD with $\alpha =.05$ and $N_{rep} = 1000$ and CDF with $N_{rep} = 5000$ and $N_{population} = 10,000$) as well as the settings that yielded the smallest bias (CD with $\alpha =.30$ and $N_{rep} = 2000$ and, again, CDF with $N_{rep} = 5000$ and $N_{population} = 10,000$). Although these settings showed the best performance, other parameter settings did not perform substantially worse (see also Tables 1 – 3). Table 4 displays the accuracy and bias of CD and CDF (using these selected hyperparameters) for different numbers of factors *k* (interested readers who want to investigate the rates of under- and overfactoring more thoroughly can find this information in additional tables in the online supplemental material). In conditions with $k=1$, CD clearly outperformed CDF (e.g., accuracy of CD with $\alpha =.05$ and $N_{rep} = 1000$ was 92.37%) and a smaller bias than CDF (e.g., accuracy of CDF with $N_{rep} = 5000$ and $N_{population} = 10,000$ was 78.27%), while in conditions with $k = 3$ CD and CDF yielded comparably high accuracies and CDF showed almost no bias, whereas CD underestimated the number of factors. In conditions with $k = 5$, CDF was clearly superior to CD as it showed a higher accuracy (85.70 vs. 78.68%) and a smaller bias (here a smaller tendency of underfactoring). Accordingly, averaged over all conditions with the same true number of factors, CD performed better in single-factor conditions, CD and CDF performed comparably well with $k = 3$, and CDF performed better with $k = 5$. The non-simulation-based EKC reached almost perfect accuracy in single-factor conditions, while being out-performed by the comparison data approaches in multi-factor cases.

Figure 3 shows the performance of both CD and CDF across conditions with different between-factor correlations ($\rho$), variables per factor (*vpf*), and numbers of factors (*k*). In data conditions with higher rates of overdetermination (i.e., more variables per factor) as well as in conditions with higher between-factor correlations, CDF yielded higher accuracies than CD, especially when $k = 5$. While CD reached a comparable accuracy in conditions with $k = 3$ and orthogonal factors ($\rho = 0$; in cases with only four variables per factor, CD had an even slightly higher accuracy), its performance decreased more with increasing between-factor correlations. The non-simulation-based EKC shows comparably high accuracy in orthogonal data conditions in general but reached substantially lower accuracy than CD or CDF in conditions with between-factor correlations. This tendency was particularly pronounced in conditions with few indicators per factor.

The loading patterns in the data generating process also had a substantial impact on accuracy.^7^ In Table 5, the accuracy of CD and CDF is displayed for all combinations of primary loading categories (small, medium, large) and cross-loading categories (zero, small, medium). CD (with $\alpha =.05$ and $N_{rep} = 1000$) outperformed CDF in conditions with large and medium-sized primary loadings (i.e., primary loadings of [0.5, 0.8]), while CDF reached higher accuracies when primary loadings were small, especially in conditions where cross-loadings were present. In conditions with small primary loadings, CD with a more liberal significance level ($\alpha =.30$) showed higher accuracy than CD with $\alpha =.05$, but was clearly inferior to CDF. EKC, for comparison, reached very high accuracy in conditions with clear simple structure patterns (i.e., independent cluster patterns without cross-loadings) and performed relatively poorly when substantive cross-loadings were present. In addition, EKC was outperformed by CDF in conditions with small primary loadings (except for simple structure conditions) reaching similar accuracy as CD.

## Discussion

In the present paper, we compared the comparison data approach with a new method called comparison data forest which combines the comparison data (Ruscio & Roche, 2012) and factor forest (Goretzko & Bühner, 2020) approaches. These findings enable us to assess which hyperparameter settings yielded higher accuracy in factor retention for each method, compare their results with regard to their accuracy and bias, derive recommendations for which method to prefer under which conditions, and suggest ideas for future research. Accordingly, the aim of this study was to refine the existing comparison data approach (Ruscio & Roche, 2012) and develop a more complex variant – the comparison data forest.

As the performance differences across CDF with different parameter settings were rather small (at least in cases with $N_{rep} \ge 1000$) and considering the computation time of the new approach, it might be a good choice to rely on CDF with $N_{rep} = 1000$ and $N_{population} = 10,000$ and use the classical CD approach (with $N_{rep} = 1000$ and $\alpha =.05$) for comparison. This choice of hyperparameters seems to be a good trade-off between accuracy and computational costs. A larger population ($N_{population}$) might be necessary when the sample size of the empirical data becomes substantially higher than the sample sizes evaluated in this study (i.e., $n>> 1000$), but for common sample sizes, $N_{population} = 10,000$ is superior.

Compared to the results of Ruscio and Roche (2012), who reported an accuracy for CD of 87.1%, in this study the overall accuracy of CD was slightly smaller (with $\alpha =.30$ and $N_{rep} = 500$ which was used by Ruscio & Roche, 2012, accuracy = 81.27%; accuracy varied from 79.74% for CD with $\alpha =.30$ and $N_{rep} = 250$ and 84.99% for CD with $\alpha =.05$ and $N_{rep} = 500$). This suggests that the conditions in our study might have been slightly more difficult, which can also explain why other hyperparameters performed best in our study ($\alpha = 0.05$ instead of $\alpha = 0.30$). Our findings that the optimal hyperparameters settings appear to be dependent on the respective data conditions may be a little unsettling for users. However, because this study was the first to systematically evaluate different hyperparameter values for CD, researchers using the approach could start with the suggested hyperparameters from this study as default values. Depending on their data conditions, a less strict $\alpha$ could be chosen to avoid underfactoring though.

**Fig. 4:**
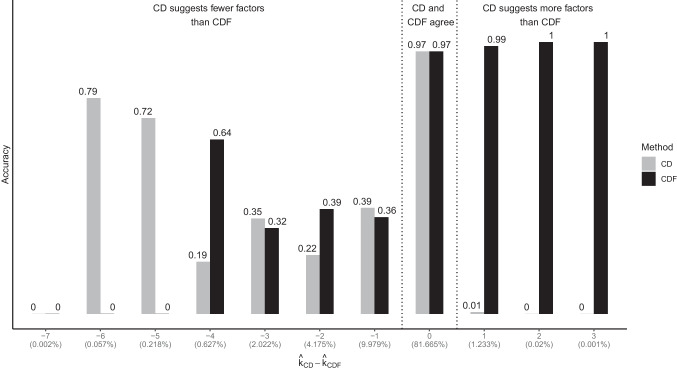
Accuracy of CD and CDF with selected hyperparameters depending on the level of their agreement ($\hat{k}_{CD} - \hat{k}_{CDF}$) [relative frequency of the scenario in brackets]

While the overall accuracy of the CDF approach was slightly higher than that of the CD approach, there were substantial performance differences in some conditions. The CD approach performed better in single-factor conditions and when primary loadings were relatively high (and hence the manifest variables were reliable indicators of the latent factors). CDF, on the other hand, was superior in conditions with five factors, with three factors and sufficient overdetermination (i.e., when there were relatively large numbers of indicators per factor), when primary loadings were small (i.e., the indicators were less reliable representations of the latent factors), when substantial between-factor correlations were present, and in small sample conditions ($N \le 500$, especially in cases with $N = 250$).

Accordingly, CD seems to be the better choice if one assumes few latent factors (unidimensionality) and/or in cases where high primary loadings can be expected (e.g., when constructing cognitive ability tests). However, if several latent variables can be expected (e.g., when developing clinically relevant personality scales) and in cases with rather small samples that are quite common in psychological research (around 50% of papers conducting an EFA have sample sizes smaller than 400, see Goretzko et al., 2019; Henson & Roberts, 2006 reported a median sample size of 267), CDF may lead to more trustworthy results. Besides, as Peterson (2000) reported that primary factor loadings were 0.32 on average (with approximately 25% smaller than 0.23) in studies presenting complete pattern matrices, it seems to be meaningful to consult CDF due to its superior performance in conditions with small loadings.

Many authors recommend that more than one factor retention criterion should be consulted when determining the number of factors in EFA (e.g., Fabrigar et al., 1999; Goretzko et al., 2019; Henson & Roberts, 2006). Along these lines, using both CD and CDF could be advisable. In the 81.7% of cases where CD and CDF agreed on the number of factors in our study, the overall accuracy of this solution was 96.6%. This is even higher (97.5%) in conditions with at least 500 observations. Therefore, when using both CD and CDF and comparing their results, agreement between methods implies a very high chance of determining the number of factors correctly.

In addition, CD and CDF may be complementary in that CD tends to underfactor (especially when *k* becomes larger) and CDF shows signs of overfactoring (especially when *k* is small). In Fig. 4, the performance of CD (with $N_{rep} = 1000$ and $\alpha =.05$) and CDF (with $N_{rep} = 5000$ and $N_{population} = 25,000$) is presented for different scenarios. Fortunately, in the 18.3% of cases where the CD and CDF methods disagreed about the number of factors to retain, we can offer some general guidelines about when to trust one method more than the other. When CDF suggests fewer factors than CD, CDF attains nearly perfect accuracy, whereas in conditions where CDF suggests way more factors than CD (mostly conditions with $k=1$ in this study), CD seems to be more trustworthy.

**Table 5:** Accuracy of CD and CDF with selected parameter settings for different loading patterns (EKC as baseline)

Method	$Acc_{small/zero}$	$Acc_{small/small}$	$Acc_{small/medium}$	$Acc_{medium/zero}$	$Acc_{medium/small}$	$Acc_{medium/medium}$	$Acc_{large/zero}$	$Acc_{large/small}$	$Acc_{large/medium}$
$CD_{.30/2000}$	0.808	0.710	0.405	0.951	0.953	0.906	0.899	0.908	0.865
$CD_{.05/1000}$	0.805	0.699	0.370	0.970	0.972	0.961	0.958	0.967	0.962
$CDF_{5000/10000}$	0.839	0.791	0.588	0.942	0.943	0.910	0.937	0.941	0.874
EKC	0.848	0.763	0.377	0.989	0.946	0.678	1.000	0.988	0.895

*Note.* $CD_{.30/2000}$ stands for the comparison data approach with $\alpha =.30$ and 2000 comparison data sets per factor solution. $CDF_{5000/10000}$ stands for the comparison data forest with 5000 comparison data sets per factor solution and a population size of 10000. EKC means empirical Kaisre criterion. $Acc_{small/zero}$ is the accuracy in conditions with small primary and zero cross-loadings, $Acc_{small/small}$ is the accuracy in conditions with small primary and small cross-loadings and so forth

The tendency of CD to underfactor can be explained by the sequential design of the method. For example, the three-factor solution is tested only if the two-factor solution attains statistically significantly better fit than the one-factor solution, so if this iterative procedure stops at any point the potentially superior fit of models with even more factors is never tested. Decreasing the $\alpha$-level of the Mann–Whitney *U* test used to check for a significant improvement in fit exacerbates this bias, and choosing a higher $\alpha$ mitigates it. On the contrary, the CDF approach simulates data for all factor solutions up to the predefined maximum number of factors (here eight). Therefore, CDF more easily suggests a higher dimensionality than CD. Although, these differences help CDF when the true number of factors is large ($k \ge 3$), they lead to a less accurate factor retention with CDF when the latent construct is unidimensional ($k=1$). Surprisingly, in single-factor conditions, CDF becomes less accurate with larger samples while factor retention criteria (including CDF in conditions with $k > 1$) usually benefit from larger samples. One explanation, again, could be that CDF simulates data for all population models from a one-factor model to a $k_{max}$-factor model. Internally, some important features for the machine learning model could be correlated to the sample size, so that larger samples are indirectly associated with a higher dimensionality within the model. Future research could investigate this rather strange behavior of the CDF approach, for example, by using interpretable machine learning methods (e.g., Molnar, 2020).

Since overfactoring often is considered less severe compared to underfactoring (e.g., Fabrigar et al., 1999), it could be reasonable to weigh the suggestion of CDF slightly more strongly than the result of CD in a combined approach. However, when combining several factor retention criteria anyway, one might also consider the results of other approaches such as parallel analysis, the empirical Kaiser criterion, or the Hull method (a combination of these methods was suggested by Auerswald & Moshagen, 2019).

Although CDF was developed as a computationally less costly alternative to the factor forest (Goretzko & Bühner, 2020), it takes substantially more time to conduct CDF than the classical CD approach. In a serial computation, CDF with $N_{rep} = 5000$ and $N_{population} = 25,000$ applied to empirical data with $N = 500$ and 35 manifest variables can take around 25–30 min depending on the computer system.^8^ This is considerably slower than other commonly used factor retention criteria. Future research could focus on improving the performance of CDF both in terms of its accuracy (e.g., introducing new features that improve the predictions of the internal random forest implementation, implementing an option that allows for hyperparameter tuning of the random forest) and with regard to its computational speed (e.g., parallelization, exclusion of features without predictive power). One promising way to develop new features that might improve the performance of CDF could be integrating other common factor retention criteria. In doing so, simulation-based approaches (such as parallel analysis) may be too computationally costly, but methods such as the minimum average partial test (MAP, Velicer, 1976) or the non-graphical scree test (Raîche, Walls, Magis, Riopel, & Blais, 2013) could be tried as features within CDF to increase its predictive performance. Future research on CDF may also evaluate its performance for various other data conditions (e.g., non-normal data, missing data, etc.) and compare it to other state-of-the-art factor retention criteria. Additional analyses (reported in the online supplemental material) indicate that CDF may provide more robust results with ordinal data (especially if the number of categories is comparably large) than common non-simulation-based methods. However, the respective results are only based on very narrow data conditions and thus have to be interpreted rather carefully. Future research should expand the simulation design in this paper and evaluate both comparison data approaches under much broader conditions that are typical for psychological research.

Older methods for determining the number of factors to retain in an EFA, such as Kaiser’s criterion or the subjective examination of a scree plot, eventually gave way to the demonstrably superior performance of parallel analysis, which entails the generation of artificial comparison data to provide reference eigenvalues. The comparison data approach builds on this to provide even more useful reference eigenvalues (by holding constant the distributions^9^ and correlations among items) and to provide sequential tests between structural models with increasing numbers of factors. The comparison data forest builds on this to take advantage of machine learning capabilities to examine even more data features when identifying the number of factors to retain. These simulation-intensive approaches enhance accuracy, are now feasible to implement in research on psychological assessment, and may be improved further by refining (through empirically tested expansion and pruning) the set of additional features to train the machine learning algorithm.

## Open Practices Statement

This article does not contain any empirical data. No preregistration was created. All R-code to run the simulation study in this paper (and to apply the new method) is presented in the supplemental material.

## Footnotes

1 This distinction between CD and parallel analysis only holds if the classic version of parallel analysis is considered. There are also implementations of parallel analysis using resampled data (i.e., permutation-based parallel analysis) that keep intact the marginal distributions of the variables. For a comparison of parallel analysis implementations, interested readers are referred to Lim and Jahng (2019).

2 In their paper, Goretzko and Bühner (2020) simulated multivariate normal data with varying sample sizes ($N \in [200,1000]$), numbers of manifest variables ($k \in [4,80]$), numbers of underlying factors ($p \in [1,8]$), different loading patterns (patterns following simple structure with varying communalities, but also complex patterns with cross-loadings and correlated factors). They used the *mvtnorm* package (Genz et al., 2018) for data simulation. We oriented ourselves to their simulation approach for comparisons (see also our “Methods” section).

3 Depending on the machine learning algorithm, some hyperparameters (parameters of the algorithm that determine how the model structure is formed – in tree-based models, for example, this could be the maximum depth a tree can grow) can be tuned in this step to increase the predictive performance of the resulting model.

4 The following features were used for the comparison data forest implementation in this study: the sample size, the number of items *p*, the number of eigenvalues of the correlation matrix that are greater than one (which is the Kaiser–Guttman rule), the relative size of each of the first three eigenvalues in a cumulative manner (i.e., the first eigenvalue alone, the first two eigenvalues together, the first three eigenvalues together), the number of eigenvalues greater than 0.7 (which is a modified variant of the Kaiser–Guttman rule that is sometimes used instead), the standard deviation of the eigenvalues, the number of eigenvalues that explain 50%/75% of indicator variance, the $L_1$-norm/ the Frobenius-norm / the maximum-norm / the spectral-norm / the determinant of the empirical correlation matrix, the number of correlations below 0.1, the average of the initial communality estimates based on the squared multiple correlations, the average of the off-diagonal correlations, the measure of sampling adequacy by Kaiser (1970), the Gini-coefficient (Gini, 1921) of the correlation matrix, the Kolm measure of inequality of the correlation matrix (Kolm, 1999), all *p* eigenvalues of the correlation matrix as well as all *p* eigenvalues of the reduced correlation matrix. We decided to rely on the full feature set of Goretzko and Bühner (2020) that has proven to result in a very accurate prediction of the number of factors (excluding only the two resource-intensive factor retention criteria; i.e., parallel analysis and comparison data).

5 Since we used comparable data conditions as Goretzko and Bühner (2020), interested readers can compare the performance of the comparison data approaches to the more cumbersome factor forest approach.

6 We analyzed all conditions that can be defined when crossing all design factors: 3 numbers of factors $\times$ 3 sample sizes $\times$ 2 numbers of variables per factor $\times$ 3 between-factor correlations $\times$ 3 different loading magnitudes for primary loadings $\times$ 3 different loading magnitudes for cross-loadings $= 486$ conditions. Excluding conditions that are not meaningful (i.e., unidimensionality and correlated factors or high primary loadings and medium-sized cross-loadings in combination with highly correlated factors and five latent variables which would yield communalities greater than one), we ended up with 372 conditions.

7 In fact, when conducting an ANOVA with all design factors as predictors (including all interaction terms) and factor retention accuracy as the dependent variable, the size of the primary loadings has the largest impact ($\hat{\eta }^2=0.183$) followed by the main effect of the cross-loadings and the second order interaction of sample size and primary loadings. A model with all interaction terms can explain roughly 80% of the variance of the factor retention accuracy, while a simpler main effects model can only explain ~40% of the variance. In comparison, a model that also includes the factor retention method as a factor (CD vs. CDF vs. EKC) explains 92% of the variance. Hence, this analysis illustrates the substantial influence of the data characteristics (i.e., the design factors of the simulation study) on the factor retention process. The size of the primary loadings appears to be especially important as its interaction with the factor retention criterion ($\hat{\eta }^2=0.015$) is the most important predictor of factor retention accuracy involving the method itself.

8 Note that this estimated run time is based on normal data conditions. For ordinal data with unfavorable item distributions, the generation of population data from which the comparison data sets are drawn can take much longer and the convergence criterion within the *GenData* function has to be adjusted to keep the run time within a reasonable range.

9 This distinction between CD and parallel analysis only holds if the classic version of parallel analysis is considered. There are also implementations of parallel analysis using resampled data (i.e., permutation-based parallel analysis) that keep intact the marginal distributions of the variables. For a comparison of parallel analysis implementations, interested readers are referred to Lim and Jahng (2019).
